# Lunar-Rhythmic Molting in Laboratory Populations of the Noble Crayfish *Astacus astacus* (Crustacea, Astacidea): An Experimental Analysis

**DOI:** 10.1371/journal.pone.0068653

**Published:** 2013-07-01

**Authors:** Robert Franke, Gabriele Hoerstgen-Schwark

**Affiliations:** Division of Aquaculture and Freshwater Ecology, Department of Animal Sciences, University of Goettingen, Goettingen, Germany; Karlsruhe Institute of Technology, Germany

## Abstract

Juvenile noble crayfish, *Astacusastacus* (Crustacea, Astacidea) in the second year of age were kept in the laboratory for a twelve-month period under continuing “summer conditions” (LD 16:8, 19°C). Molting processes in this population could be synchronized by artificial moonlight cycles. Peaks of exuviations occurred at “new moons”. Males showed a slightly higher degree of synchronization than females. A phase-shift of the artificial lunar cycle in relation to the natural cycle resulted in a corresponding shift of the molting cycle. This clearly demonstrates that changes in the nocturnal light regime provide the primary external information for the lunar-monthly molting rhythm. There is a first indication that lunar photic stimuli do not act directly but as a zeitgeber which entrains an endogenous molting rhythm to the lunar cycle. Moreover, the results of the long-term experiments suggest that the hibernal resting period of *A* *. astacus* in the field (no molts between October and April) may also involve some endogenous programming. Continuing artificial summer conditions can delay but not completely suppress this resting period. The adaptive significance of the phenomena and how the findings may be applied to improve the management of crowded crayfish stocks are discussed.

## Introduction

Most crustaceans molt throughout their lifetime. The timing of molting events is influenced by a variety of internal and external factors [1]. Molting is often coupled to distinct phases of geophysical cycles. Seasonal synchrony of molting is well known for crustaceans of higher latitudes. However, molts can also be coupled with environmental cycles of shorter periodicities such as the lunar-monthly cycle, the semi-lunar cycle of spring-neap tides, the daily cycle, and the tidal cycle. This has been reported particularly for a number of marine crustaceans [2–9] and some insects [10,11]. The adaptive significance of these phenomena as well as the underlying mechanisms are only little understood.

Freshwater crayfish usually favor a particular time of day for molting, although there is no uniform pattern among species: nocturnal molting is known for *Cherax* *destructor* [12], while *Procambarus* *clarkii* [13] and *Astacusastacus* [14] molt mainly during daytime. As to a possible coupling of molting to lunar cycles, no information was available for any species of freshwater crayfish until recently. However, results reported in a recent paper [14] suggest that molting processes in a laboratory population of the noble crayfish *A* *. astacus* can be synchronized by an artificial moonlight cycle.

The coupling of molting processes to certain phases of the lunar cycle requires a periodic input of relevant external information. The moon’s rotation relative to the earth is accompanied by a variety of regular environmental changes [15]. The most obvious among these changes relate to gravitational forces and to the lunar-monthly changes in the nocturnal light regime (moonlight cycle). Additionally, a number of subtle geophysical parameters such as electromagnetic forces also undergo lunar-monthly changes and hence have also to be considered as potential sources of external information [16]. Only stringent experimental analyses can specify which particular environmental variables are involved in a lunar-rhythmic timing of molting or other activities.

Effects of moonlight on life on earth have been a matter of speculation over centuries. Since the 1960s, however, unequivocal evidence has been provided for a role of moonlight in a number of different contexts: a) orientation and navigation at night [17–19]; b) regulation of nocturnal activity [20,21]; c) generation of lunar rhythms of reproduction, molting or other activities [22–26].

Environmental factors can operate either directly (exogenous control) or indirectly (endogenous control) in the generation of biological rhythms that are linked to geophysical cycles. Exogenously controlled rhythms depend on a continuous input of certain external stimuli. Endogenously controlled rhythms are generated by an internal clock mechanism, and external factors merely act as entraining agents (zeitgebers) which adjust (entrain) the internal clock to a geophysical cycle of similar period length [27,28]. In this case, the biological rhythm would persist at least for some time when shielded from the relevant external synchronizer (“constant conditions”), showing under these conditions its spontaneous (free-running) period which closely matches, but slightly deviates from that of the environmental cycle. Although this model has been developed and tested primarily for daily (circadian) rhythms, it may also apply to other rhythms (circatidal, circalunar and circannual) which are in line with geophysical cycles [5,11,29,30].

The present paper deals with the following topics: (1) Our preliminary findings on the synchronization of molting processes in *A* *. astacus* by an artificial moonlight cycle [14] were re-examined on a larger experimental basis, and the data were analyzed with respect to possible sex-specific differences in the animals’ responses to moonlight. (2) Furthermore, an experiment was conducted to produce conclusive evidence (or counterevidence) of the moonlight cycle providing the primary source of environmental information which noble crayfish use to couple molting events to a particular phase of the lunar cycle. For that purpose the daily patterns of molt frequencies were studied under artificial moonlight cycles which were phase-shifted against each other by half the lunar period(3). Another experiment was performed to gain first insight into whether the lunar molting rhythm is exogenously or endogenously controlled. Laboratory populations of *A* *. astacus* were first synchronized by artificial moonlight cycles, but then released from any further lunar-rhythmic photic stimuli to test for the occurrence of free-running circa-lunar molting rhythms(4). Finally, possible adaptive values of the observed phenomena and how the findings can be used to improve crayfish broodstock management in indoor-recirculation systems are discussed.

## Materials and Methods

### Ethics Statement

The study conforms to German laws and did not require any permissions.

### Animal material and culture conditions

The animal material originated from a German crayfish farm (Helmut Jeske, Oeversee). For the experiments the crayfish were kept in single-sex groups of 80 (experiment 1) or 100 (experiment 2) individuals each in shallow tanks (1.4 m^2^, water volume 165L) which were elements of an established indoor recirculation system (for details [14]). The tanks were equipped with a surplus of plastic tubes as hiding places. A highly diversified diet was offered daily in the evening (oat flakes, commercial pellets of mixed plant and animal origin, and various kinds of defrosted food: chironomid larvae, flour worms, fish, spinach), and unconsumed food was removed in the morning.

Water temperature was constant at 19 ± 1°C. The tanks were located in lightproof rooms shielded from natural light. A constant long-day light regime of LD 16:8 was applied using fluorescent lamps (Philips Master TL-D 865, color temperature 6500 K, spectrum rich in short and medium wavelengths). Light intensity at the water surface during the day was constant but ranged between 110–350 lx over the area of the large tanks. The moonlight cycle was simulated by a dim artificial light (tungsten filament lamp, full spectrum) to which the animals were exposed during a sequence of six nights every lunar month (period of “full moon”). The intensity of this light at the water surface was 1.5 to 2.5 lx which is slightly higher than, but roughly corresponds to, the intensity of the natural full moon (about 0.3-0.5 lx). All other nights of the lunar cycle were absolutely dark. Light intensity (illuminance) was measured with a photometer (Photo-Meter 1, Quantum Instruments Inc.).

During the experiments all tanks were checked daily for molts. Exuviae were removed to avoid double counts.

### Experiment 1

At the start of the experiment (November 11, 2011), the animals (160 males, carapace length 35-40 mm; and 160 females, carapace length 30-35 mm) were about 18 months old and had already been kept under constant ”summer conditions“ (19 ± 1°C, LD 16:8) and an artificial moonlight cycle for more than six months (since April 2011). The moonlight cycle was in phase with the natural lunar cycle, i.e. the six successive moonlit nights were symmetrically distributed around the days of natural full moon (nights between days 13 and 19 of the lunar cycle, see below). Molt frequencies were recorded daily, separately for the two sexes, over three complete lunar cycles from November 11, 2011 (day after full moon) to February 07, 2012 (full moon).

### Experiment 2

The animals of Experiment 2 were taken out of outdoor ponds of a crayfish farm in mid-March 2012. In these ponds the crayfish had hatched in summer 2011 and had grown up there under natural environmental conditions. With the decreasing water temperature in October, animals were prevented from further molts and thus growth. Under natural conditions this hibernal break in molting processes would have continued as far as May. When transferred to the laboratory (March 2012), the crayfish were thus about eight months old; their carapace length ranged between 10 and 15 mm in both males and females. In the laboratory the crayfish were acclimated to the standard conditions (19 ± 1°C, LD 16:8) within two weeks. At the start of the experiment (March 30, 2012) the animals were allocated to two groups (A, B). Each group consisted of 200 crayfish (100 males and 100 females, kept in separate tanks). Animals of group A were exposed to an artificial moonlight cycle which was in phase with the natural cycle (same design as in Experiment 1). Animals of group B were kept under identical conditions with a single exception: The artificial moonlight cycle was phase-shifted by half the lunar period, i.e. artificial moonlight was given the three nights before and after the days of natural new moon (corresponding to the nights between days 28 and 4 of the lunar cycle). Molt frequencies were recorded daily over a one-year period from March 30, 2012 to March 29, 2013. On December 03, 2012 the artificial moonlight cycles were switched off for both groups, i.e. from that date on animals did no longer receive lunar-periodic photic stimuli (all nights absolutely dark).

### Statistical analyses

To reveal and analyze periodicities in the time series of daily molting frequencies, data were de-trended and subjected to spectral (Fourier-) analyses (software STATISTICA 10.0, StatSoft^©^). Spectral densities were calculated by smoothing periodogram values (Hamming data window, width 5) to identify frequency areas which significantly contribute to the periodic character of the time series. Bartlett’s Kolmogoroff-Smirnov statistics (Bartlett K–S d) was applied to test the null hypothesis (1% significance level) that the time series is a product of white noise.

The distributions of molting frequencies around the lunar cycle were analyzed with the methods of circular statistics [31] using the software Oriana 4.0 (Kovach Computer Services). For the circular representation of the data, calendar days were transformed into lunar days (1-30) with day 1 and 16 representing the days of natural new and full moon, respectively. Lunar days were furthermore converted into 30 phase angle groups of 12° each, with group 0° (range: 354°-6°) corresponding to day 1 (new moon) and group 180° (range: 174°-186°) corresponding to day 16 (full moon). The other 28 groups represent the seven days before and after new moon and full moon, respectively.

Molt frequency data followed a von Mises distribution (“circular normal” distribution). The mean angular direction (mean lunar day of molting, with 95% confidence interval) was calculated, indicating the phase of the moon in which molting was concentrated. To estimate the dispersion of molting dates over the lunar cycle, the angular standard deviation (s) was calculated. The null hypothesis that molts were randomly distributed over the lunar cycle was tested with the Rayleigh test. Additionally, a circular-linear correlation analysis was performed to test for a significant relationship between daily molt frequencies and the lunar cycle. Possible unspecified differences (differences in mean angular direction and/or in mean angular deviation) between the sexes were analyzed with the Mardia-Watson-Wheeler test. For this purpose ties between samples were broken by random allocation. A parametric test for the concentration parameter was performed to analyze whether males and females differ in the mean angular deviation of their lunar molting dates [32].

## Results

### Experiment 1

**Figure 1:**
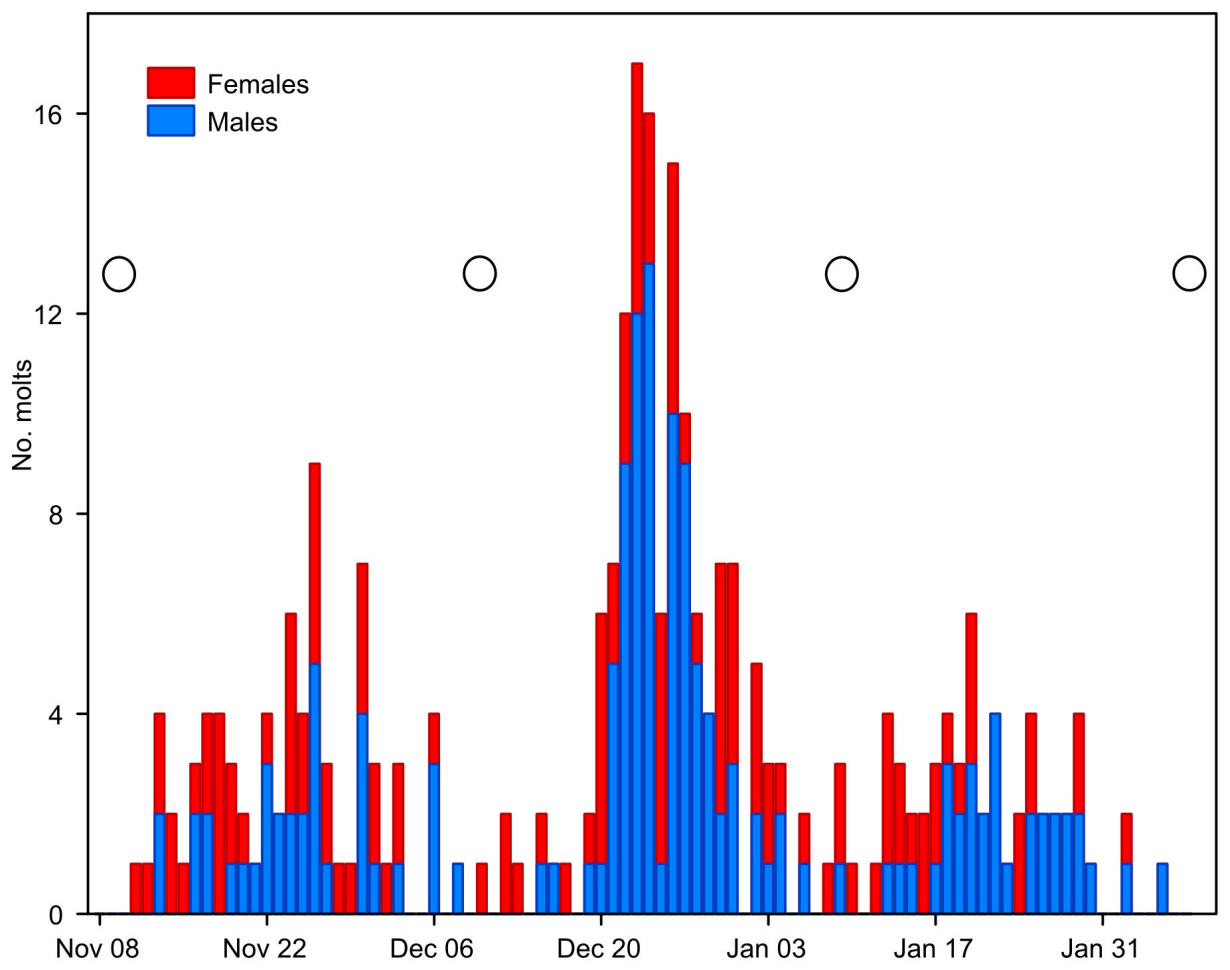
Daily molt frequencies of males and females over the three-month experimental period (experiment 1). Stacked bar plot; days of full moon indicated by open circles.

**Figure 2:**
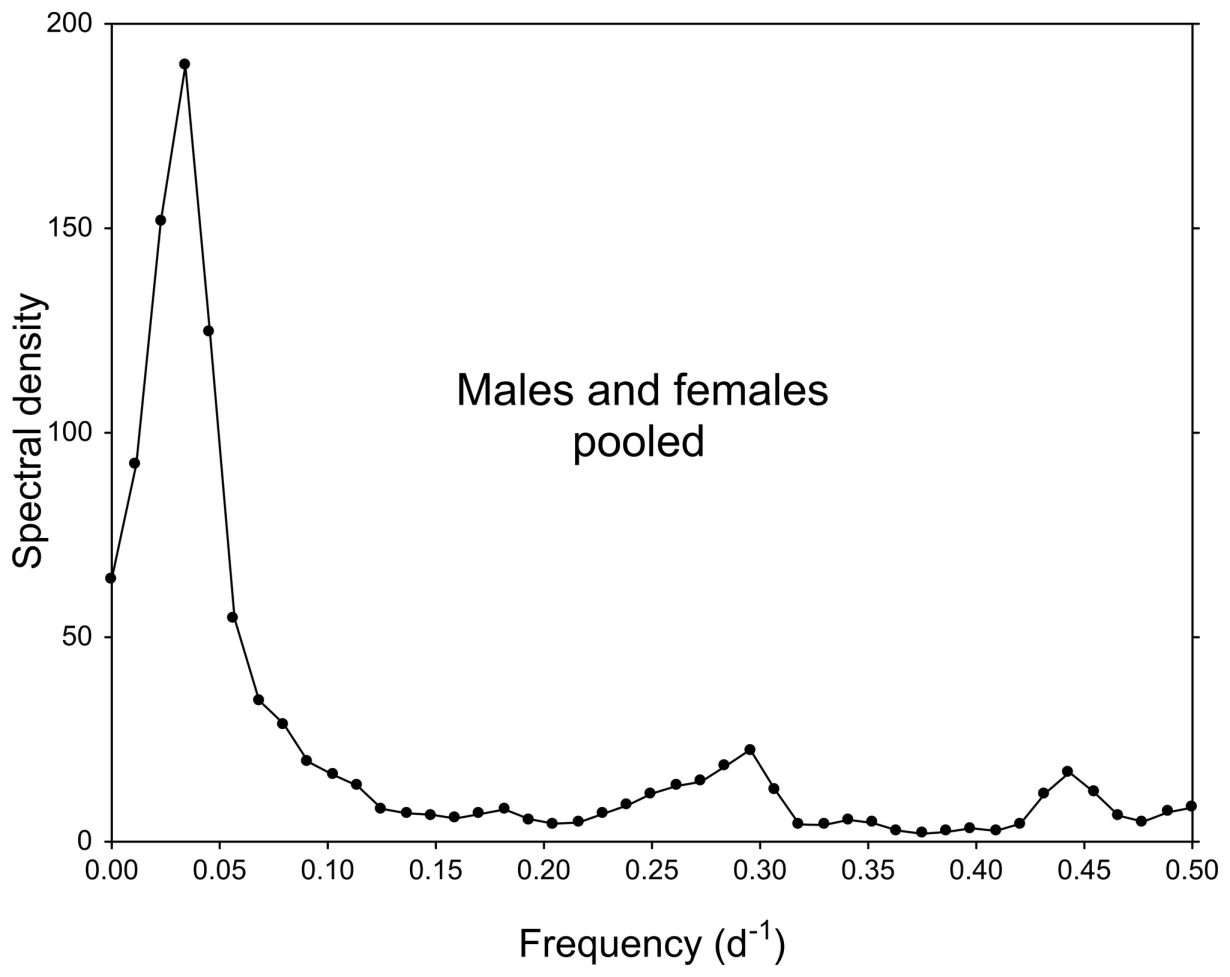
Spectral density plot for the three-month time series of daily molt frequencies (experiment 1). Males and females pooled; peak at 0.034 d^-1^.

The mortality rates over the experimental period (three months) were insignificant (5% for males and 8% for females). A total of 274 molts were recorded (152 males, 122 females). Daily molting frequencies plotted over the complete three-month period (Nov 11, 2011-Feb 07, 2012) show a clear lunar-monthly periodicity in both males and females (Figure 1). The spectral density plot (Figure 2) for this time series exhibits a single conspicuous peak at a frequency of about 0.034 d^-1^, corresponding to a period of about 29.3 d. Oscillations in this frequency/period range thus make the most substantial contribution to the observed periodicity in the time series. The time series departs significantly from white noise: Bartlett K–S d = 0.5680 > critical value (m = 92) = 0.1709.

**Figure 3:**
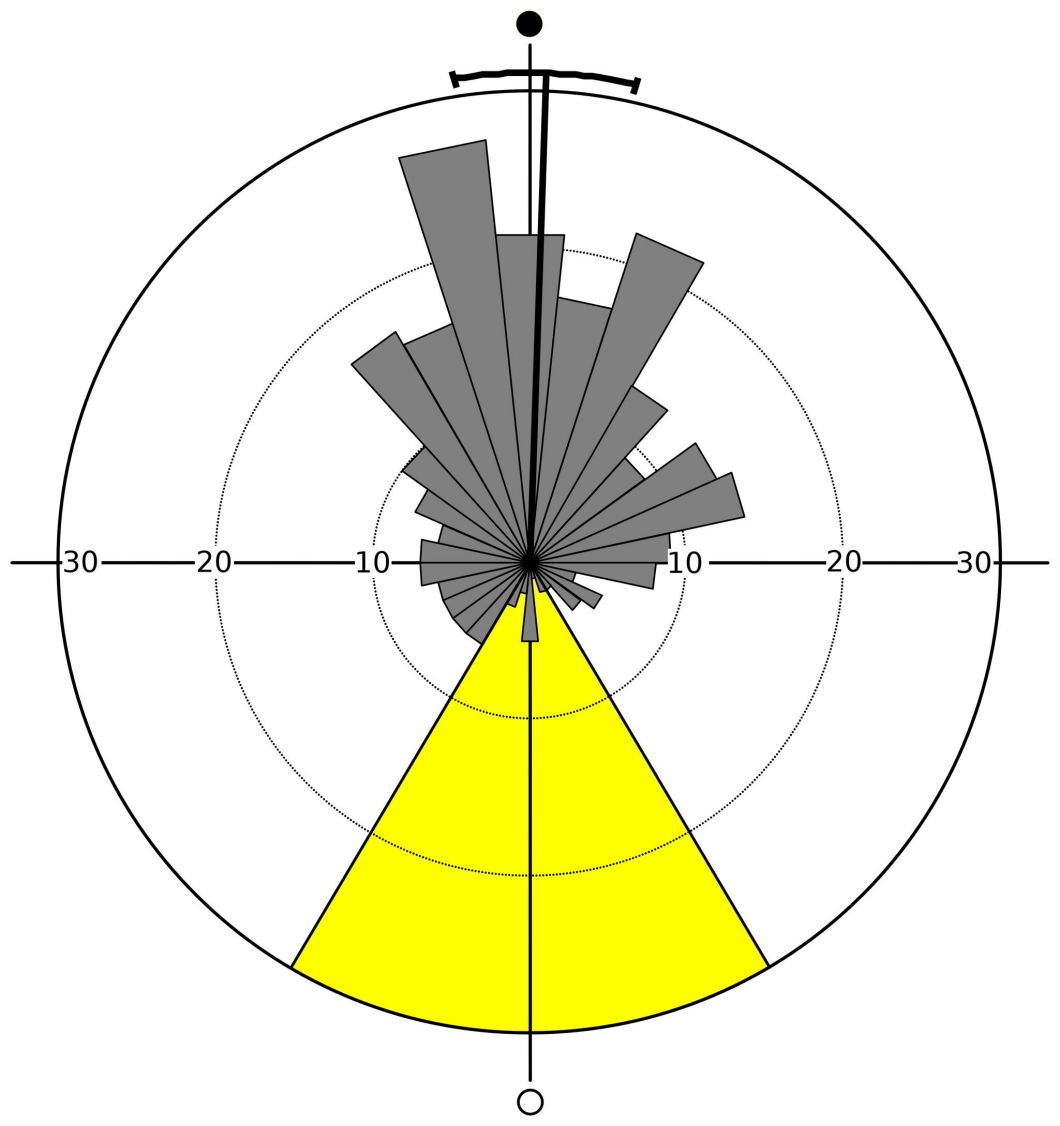
Circular plot for daily molt frequencies over the lunar cycle (experiment 1) - pooled data for males and females (N = 274). Mean angular direction and its 95% confidence interval are indicated. Open circle = natural full moon, filled circle = natural new moon; period of exposure to artificial moonlight (three nights before and after the day of full moon) indicated in yellow.

Although molting events were dispersed over the complete lunar cycle, frequencies clearly peaked around new moon (Figure 3). The mean angular direction was 1.9° (95% confidence interval: 351.4°-12.5°) which corresponds to the day of new moon (day 1). The circular standard deviation was 74.3°. Rayleigh’s test clearly showed that the distribution was significantly different from random (Z = 51.2, p < 0.01). Furthermore, daily molt frequencies exhibited a significant circular-linear correlation with the lunar cycle (r = 0.86, p < 0.01).

**Figure 4:**
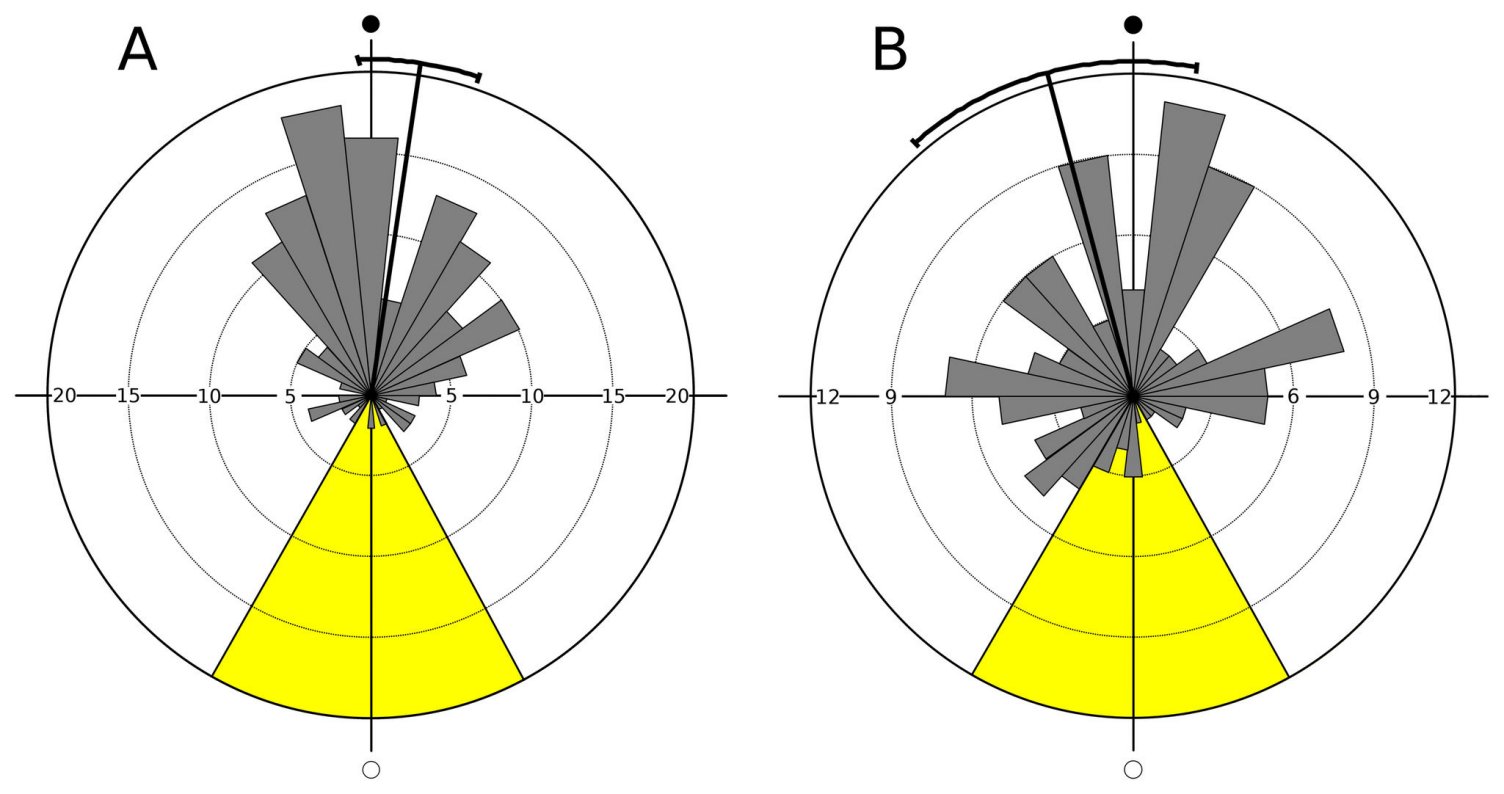
Circular plot for daily molt frequencies over the lunar cycle (experiment 1) - calculated separately for males (A, N = 152) and females (B, N = 122). Mean angular direction and its 95% confidence interval are indicated. Open circle = natural full moon, filled circle = natural new moon; period of exposure to artificial moonlight (three nights before and after the day of full moon) indicated in yellow.

The mean angular directions calculated separately for the two sexes (Figure 4), were 8.4° (95% confidence interval: 358.0°-18.7°) for males (day after new moon) and 345.2° (319.4°-10.9°) for females (day before new moon). The circular standard deviations amounted to 61.0° (males) and 92.2° (females). The results of the Rayleigh test revealed non-random distributions of molting frequencies in both males (Z = 48.9, p > 0.01) and females (Z = 9.1, p < 0.01). The circular-linear correlations with the lunar cycle were significant in both males (r = 0.82, p < 0.01) and females (r = 0.60, p < 0.01). The Mardia-Watson-Wheeler test showed a significant unspecified difference between males and females (W = 14.9, p < 0.01). The subsequent parametric test indicated that this was due to a significant difference in mean angular deviation (F = 1.47, n_1_ = 151, n_2_ = 121, p < 0.01), while no such difference was found with respect to the mean angular direction (mean lunar day of molting). Molting events in females were thus significantly less well synchronized with the lunar cycle than those in males.

### Experiment 2

**Figure 5:**
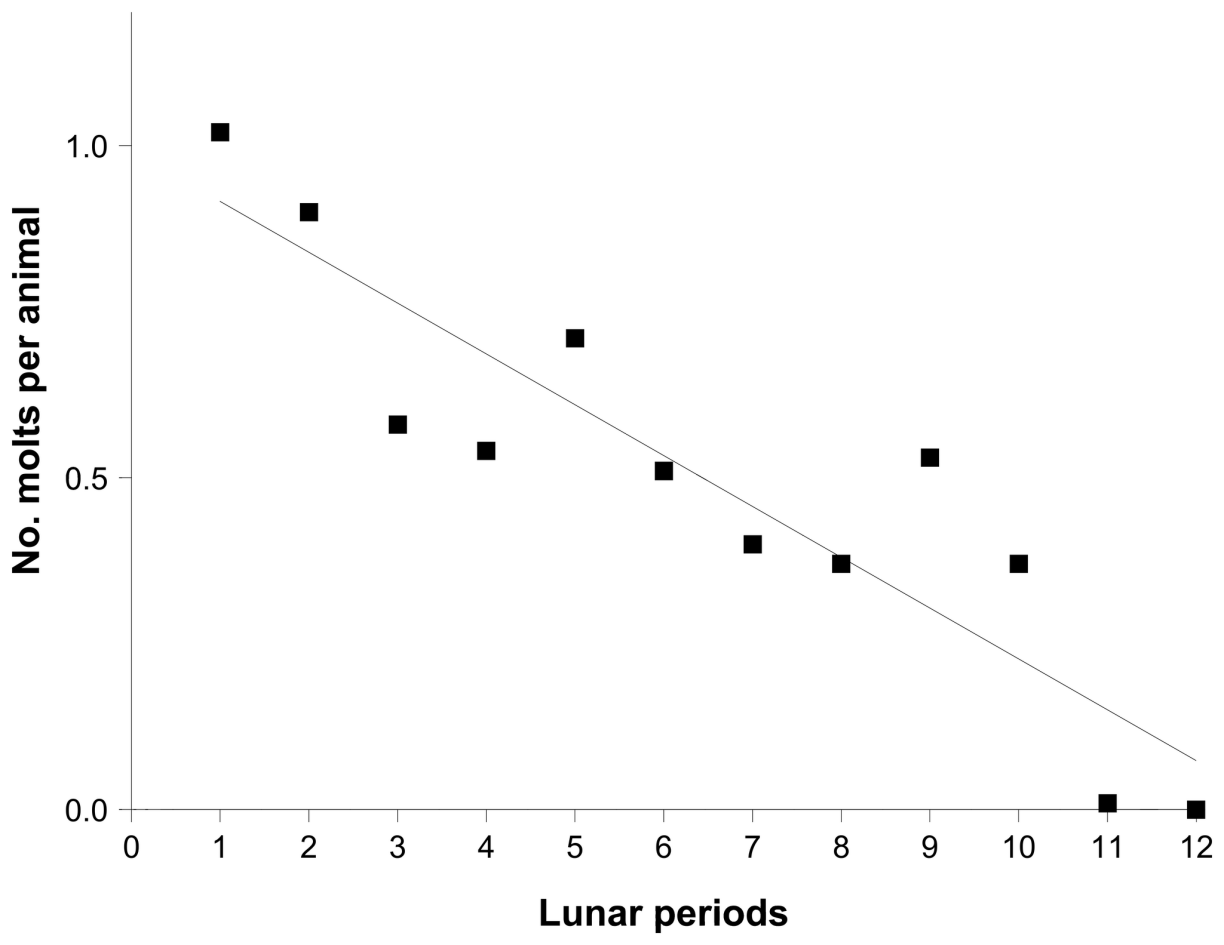
Number of molts per animal during the successive lunar cycles of the one-year experimental period (experiment 2). Linear regression line is shown.

The mortality rate over the complete one-year experimental period was only 12% (males: 16%, females: 8%). A total of 2,220 molts were registered (1,108 males and 1,112 females). The number of molts per animal and lunar cycle decreased significantly over time (linear regression: r^2^ = 0.823, p < 0.0001), reflecting an increase in mean molt interval (intermolt period) with age (Figure 5).

**Figure 6:**
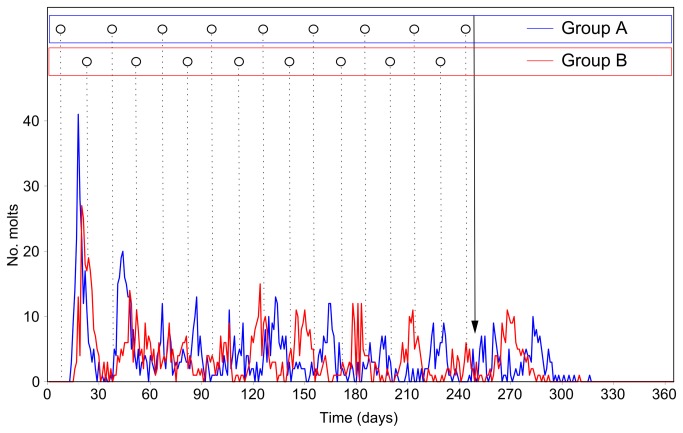
Daily molt frequencies over a one year period under artificial moonlight cycles (experiment 2). Open circles indicate full moons; day 1 = March 30, 2012; day 365 = March 29, 2013. Group A (blue): artificial cycle in phase with the natural lunar cycle; Group B (red): artificial cycle phase-shifted by half the lunar period. Arrow: Termination of moonlight regimes on December 03 (Day 249).

The rapid increase in temperature immediately prior to the start of the experiment represented a strong stimulus which terminated the hibernal break of molting processes. Almost all crayfish (392 out of 400) molted rather synchronously within about four weeks (Figure 6). Thereafter, lunar-monthly changes in daily molt frequencies gradually established in both groups (and independent of sex). The rhythms of Groups A and B were clearly phase-shifted against each other by about half the lunar period, reflecting the phase-shift of the artificial moonlight cycles which animals of groups A and B were exposed to.

**Figure 7:**
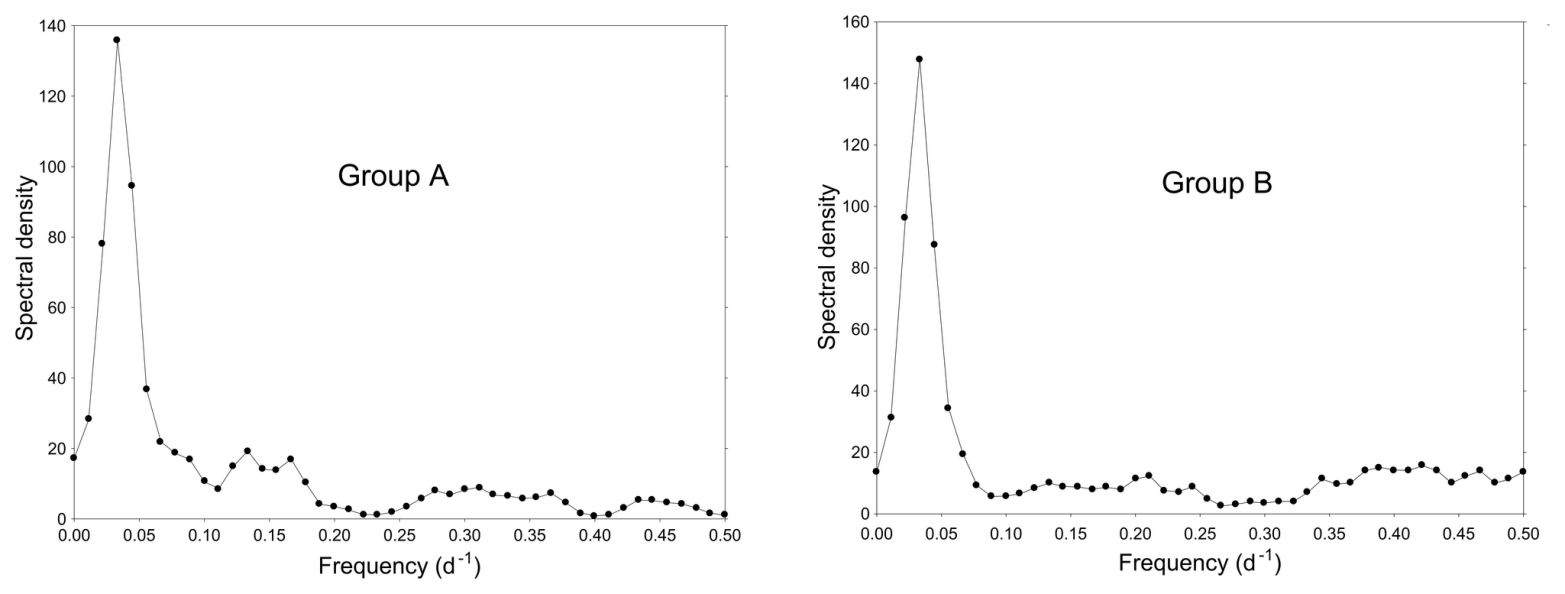
Spectral density plots for the three-month time series of daily molt frequencies (experiment 2). Males and females pooled. Group A (artificial cycle in phase with the natural lunar cycle) with peak at 0.033 d^-1^ and group B (artificial cycle phase-shifted by half the lunar period) with peak at 0.033 d^-1^.

Referring to the procedure in Experiment 1, data from a period of three complete lunar cycles (September 01 to November 28, 2012) were statistically analyzed. A total of 244 (group A) and 232 (group B) molts were recorded over this period of time. For both groups, spectral density plots show a single conspicuous peak at a frequency of 0.033 d^-1^, corresponding to a period of 30.0 d (Figure 7). Oscillations with frequencies/periods around these values thus contributed most to the observed rhythmic character of the time series. Both time series depart significantly from white noise: Bartlett K–S d = 0.5680 > critical value (m = 90) = 0.1728 for group A, and Bartlett K–S d = 0.4357 > critical value (m = 90) = 0.1728 for group B.

**Figure 8:**
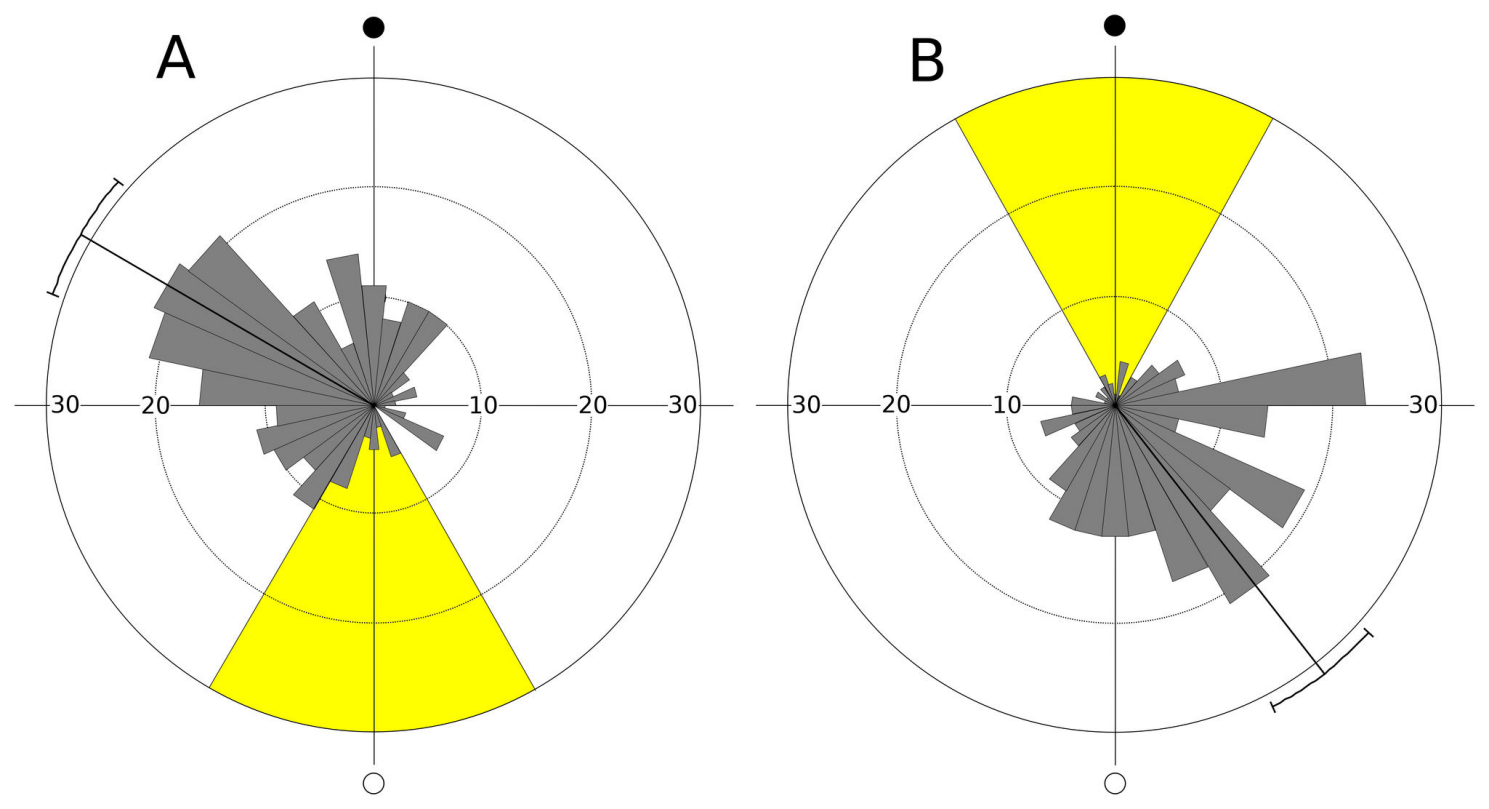
Circular plots for daily molt frequencies over the lunar cycle (experiment 2). Group A (N = 244): artificial cycle in phase with the natural lunar cycle; group B (N = 232): artificial cycle phase-shifted by half the lunar period. Pooled data for males and females. Mean angular direction and its 95% confidence interval are indicated. Open circle = natural full moon, filled circle = natural new moon; period of exposure to artificial moonlight marked in yellow.

The mean angular direction of molting in group A (males and females pooled) was 300.1° (95% confidence interval: 289.1°-311.2°) which corresponds to day 26 of both the natural and the artificial lunar cycle (Figure 8). The circular standard deviation was 73.7°. The distribution was significantly different from random (Rayleigh test: Z = 46.6, p < 0.01). Daily molt frequencies showed a significant circular-linear correlation with the lunar cycle (r = 0.84, p < 0.01). The mean angular direction of molting in group B was 142.1° (95% confidence interval: 131.9°-152.3°) which corresponds to day 13 of the natural and day 28 of the artificial lunar cycle, respectively (Figure 8). The circular standard deviation was 73.7°. While groups A and B differed significantly in the mean direction of molting, no difference between the groups was found in the concentration parameter (test according to [32], p. 161).

After the artificial moonlight cycles had been switched off (Dec 03, 2012), only a few further peaks in daily molting frequencies occurred (one in Group B and two in Group A), continuing (in the absence of lunar-periodic photic cues) the rhythms which had established previously under the influence of artificial moonlight cycles. Surprisingly, however, the molting rhythms in both groups did not fade out, but came to an abrupt end. Virtually no further molts were recorded from mid-January, 2013 till the end of the experiment (March 29, 2013) exactly one year after it had been started. Due to the lack of further molts, it was impossible to calculate a free-running period of the molting rhythm.

## Discussion

Molt intervals in *A* *. astacus* increase strongly with age. During their first summer, young crayfish molt up to six times, corresponding to molt intervals between about 10 and 30 days [33,34]. In the second summer molt intervals are already longer than a month and this trend continues up to the individual’s death. At the individual level, molting is hence not rhythmic in a strict sense. Nevertheless, when coupled to particular phases of environmental cycles, processes which are arrhythmic at the individual level or even occur only once in an individual’s life time can display a marked rhythm at the level of the population. This also applies to the observed molting rhythm in *A* *. astacus* which manifests only at the population level.

Under an artificial moonlight cycle, molting processes in a laboratory population of *A* *. astacus* (age of 18 months and more) showed a clear rhythmic component, with a period corresponding to that of the moonlight cycle (Experiment 1). Peaks of molting coincided with “new moons”, i.e. with the days in the middle between successive periods of moonlit nights. The degree of molting synchrony was higher in males than in females, whereas period and phasing did not differ between the sexes.

The design of Experiment 1 (artificial moonlight cycle in phase with the natural cycle) does not allow for a conclusive evidence that it is actually photic stimuli which couple molting processes to a particular phase of the lunar cycle. This is because the controlled laboratory conditions were inevitably pervaded by periodic changes of some subtle geophysical parameters (e.g. electromagnetic fields) which are related to the moon’s rotation around the earth. There are a number of findings suggesting that such factors can be perceived by organisms [16,35,36] and thus must not be ignored as potential sources of external information.

The results of Experiment 2, however, clearly show that the primary external information for the observed lunar periodicity in molting originates from the lunar-monthly changes in the nocturnal light regime. A shift of the periods of artificial full moon into the natural new moons resulted in a corresponding phase shift in the molting rhythm of rather the same magnitude (mean lunar day of molting). Furthermore, the degree of molting synchrony (mean angular deviation of molting) was independent of whether the artificial full moons coincided with full moons or else with new moons of the natural cycle. If other lunar-rhythmic factors are involved at all, there is a clear predominance of photic information over other cues.

Surprisingly, the phase-relationship between the artificial moonlight cycle and the molting rhythm in Group A of Experiment 2 (mean lunar day of molting: day 26) differed slightly but significantly (Mardia-Watson-Wheeler Test: W = 51.3, p < 0.01) from that observed in Experiment 1 (mean lunar day of molting: day 1), although in both set-ups the artificial moonlight cycle was in phase with the natural cycle (Figures 3 and 8). As is known for circadian rhythms, several external and internal factors influence the phase-relationship between environmental cues and the overt biological rhythm [37]. The phase-relation between lunar cues and the peak of exuviations in *A* *. astacus* may thus be altered by external factors such as temperature, number of moonlit nights per cycle, and moonlight intensity as well as by internal factors such as age/size, previous history, physiological status etc. As the external parameters in both experimental set-ups were largely unchanged, the recorded slight difference in phase-relationship may have resulted from differences in the animals’ internal conditions such as age and previous history.

Coupling of biological rhythms with the lunar cycle via photic stimuli associated with the moonlight cycle have been suggested for a number of marine, freshwater and terrestrial species, yet experimental evidence is still restricted to a few cases (for reviews, see 26,38,39). In most cases studied, photic lunar cues do not seem to act directly but as zeitgebers (entraining agents) of an endogenous (semi-) lunar rhythm. Irregularities in weather conditions make moonlight a rather unreliable cue in many parts of the world. An endogenous nature would allow for the maintenance of the rhythm even when the moon is occasionally obscured by clouds.

The available data from Experiment 2 do not allow for a clear statement on whether the observed lunar rhythm is exogenously or endogenously controlled. The few further peaks which occurred after the synchronized populations (Groups A and B) had been released from lunar photic stimuli, represent a first indication of an endogenous nature of the rhythm. However, due to the failure of further molts, it was not possible to demonstrate a free-running rhythm and to calculate its period. The nearly complete absence of molts from mid-January 2013 can hardly be attributed to the missing moonlight cycle. From March 2012, the experimental animals had been maintained under continuing “summer conditions”. Under ambient conditions, these animals would have stopped growing and molting by October at the latest, resuming these processes not before late spring of the following year. Our laboratory findings suggest that such a hibernal resting period is not merely a direct response to declining environmental conditions. Some endogenous programming seems to be involved. Maintaining crayfish under continuing summer conditions may delay its onset by several months into January, but cannot suppress it completely in the long run. The question whether there is an endogenously programmed seasonal resting period in juvenile and adult *A* *. astacus* is of great relevance to the management of broodstocks as crayfish production in indoor recirculation systems would greatly benefit from a continuous growth of the stock, uncoupled from the seasonal cycle. This problem deserves a detailed experimental analysis which was beyond the scope of the present paper.

What selective advantage(s) do crayfish derive by synchronizing their molts (exuviations) with the days around new moon? The act of exuviation is a crucial event. At and immediately after exuviations, crayfish probably are vulnerable targets to predators and exposed to an increased danger of cannibalistic attacks. Benefits may arise in two different contexts which are not mutually exclusive: (1) Molts are timed to a distinct phase of the lunar cycle, as the environmental conditions at this point of time are more appropriate to successful molts than they are at other times. (2) Molting at the same time may provide some advantage to all individuals involved. In this case, synchronization with the external cycle would be but a devious route by which animals of a population achieve some interindividual synchrony.

*Astacusastacus* may derive benefits in both of the contexts mentioned above: 1) The species is nocturnally active, and so are its main predators ( *Anguilla* *anguilla* , *Sander* *lucioperca* , *Silurus* *glanis* ). Many nocturnally active prey species are known to reduce their activity during the bright nights around full moon [40]. This has often been related to predator–prey interactions [41,42]. Although primarily oriented by the senses of smell and/or touch, crepuscular and nocturnal predators are often highly sensitive to light which would expose nocturnal prey species to an increased risk of visual detection during moonlit nights. Reducing the level of activity around full moon may thus be interpreted as a predator avoidance response. The same argument may apply to the coupling of molts to the days around new moon in *A* *. astacus* : The avoidance of molts around full moon may have been selected to reduce mortality by predators and cannibalistic conspecifics. 2) Spatial clustering (e.g. the formation of fish schools) is regarded as an adaptation by which animals reduce their individual risk of predation (selfish herd hypothesis [43]). A similar effect may result from a temporal clustering as suggested by Reaka [3] to explain the lunar coupling of molting in some mantis shrimps (Stomatopoda). If a large number of crayfish molt at the same time, a temporal excess of freshly molted animals would dilute the effects of predation and cannibalism. Cannibalism towards freshly molted animals is the main cause of mortality in crowded stocks of *A* *. astacus* [44–47]. Reducing the losses by cannibalism is a basic requirement for a successful intensive crayfish production. The application of an artificial moonlight cycle may help to improve the management of crayfish stocks particularly in indoor recirculation systems. In this context it would be advantageous to increase the degree of interindividual synchrony in molting beyond that observed in the present study. Further experiments have to show whether this can be achieved by (a) changing (e.g. increasing) the intensity of moonlight, and/or (b) changing (e.g. reducing) the number of moonlit nights per lunar cycle.
